# Multimodality analysis of Hyper-reflective Foci and Hard Exudates in Patients with Diabetic Retinopathy

**DOI:** 10.1038/s41598-017-01733-0

**Published:** 2017-05-08

**Authors:** Sijie Niu, Chenchen Yu, Qiang Chen, Songtao Yuan, Jiang Lin, Wen Fan, Qinghuai Liu

**Affiliations:** 1 School of Information Science and Engineering, University of Jinan, Jinan, 250022 China; 2 School of Computer Science and Engineering, Nanjing University of Science and Technology, Nanjing, 210094 China; 3 Department of Ophthalmology, The First Affiliated Hospital with Nanjing Medical University, Nanjing, 210094 China; 4 Department of Endocrinology, Nanjing Medical University, Nanjing, 210094 China

## Abstract

To investigate the correlations between hyper-reflective foci and hard exudates in patients with non-proliferative diabetic retinopathy (NPDR) and proliferative diabetic retinopathy (PDR) by spectral-domain optical coherence tomography (SD OCT) images. Hyper-reflective foci in retinal SD OCT images were automatically detected by the developed algorithm. Then, the cropped CFP images generated by the semi-automatic registration method were automatically segmented for the hard exudates and corrected by the experienced clinical ophthalmologist. Finally, a set of 5 quantitative imaging features were automatically extracted from SD OCT images, which were used for investigating the correlations of hyper-reflective foci and hard exudates and predicting the severity of diabetic retinopathy. Experimental results demonstrated the positive correlations in area and amount between hard exudates and hyper-reflective foci at different stages of diabetic retinopathy, with statistical significance (all p < 0.05). In addition, the area and amount can be taken as potential discriminant indicators of the severity of diabetic retinopathy.

## Introduction

Diabetic retinopathy (DR), a common microvascular complication of diabetes, remains the leading cause of vision loss in many developed countries and continues to increase in developing countries^1–3^. Apart from its effects on vision, the presence of diabetic retinopathy also signifies a heightened risk of life-threatening systemic vascular complications^4^. Diabetic retinopathy is normally categorized into two main types: non-proliferative diabetic retinopathy (NPDR) and proliferative diabetic retinopathy (PDR). In both NPDR and PDR, the breakdown of the blood-retinal barrier often causes retinal changes such as hemorrhages, hard exudates (HEs), and hyper-reflective foci (HRF), which may lead to decreased vision^5^. HRF are a morphological sign of accumulation of lipid extravasation, proteinaceous material and inflammatory cells, and consequently precursors of HEs^6, 7^. They are deposited primarily in the outer plexiform layer (OPL) and outer nuclear layer (ONL) of the retina^6–8^. Analyzing the retinal changes in the diabetic retinopathy is helpful to determine the risk of vision loss.

Retinal imaging is widely used by ophthalmologists to screen for epidemic retinal diseases. Color fundus photography (CFP) has been taken as the gold standard in the diagnosis of retinal diseases. With the development of retinal imaging, optical coherence tomography (OCT) has become a popular noninvasive optical imaging modality used to detect pathologic changes^9–14^, objectively measures the macular retinal thickness in patients affected by DR^15^, and tracks the progression of retinal diseases^16, 17^, in particular spectral domain optical coherence tomography (SD OCT). Compared with retinal changes two-dimensionally provided by CFP, SD OCT offers three-dimensional information about the extent and distribution of hyper-reflective lesions throughout the retinal layers, enabling the visualization of HRF.

Recently, hard exudates and hyper-reflective foci in retinal diseases have been widely studied to analyze the correlation with vision loss. Several studies have performed analysis to determine the hyper-reflective foci or hard exudate associated with diabetic macular edema (DME) using OCT images. De Benedetto *et al*.^18^ reported that SD OCT findings may be a useful marker for the diagnosis and follow-up in the early stages of diabetic retinopathy. Lammer *et al*.^19^ analyzed HEs and their precursors in patients with DME by using polarization-sensitive OCT (PS OCT). They concluded PS OCT may provide a novel method for assessing and quantifying HEs. Additionally, in some cases, these HRF may be seen without any corresponding hard exudates^19^. Ota *et al*.^20^ demonstrated subretinal HRF in patients with DME may be associated with the future subfoveal deposition of HEs. Deák *et al*.^21^ suggested that HRF in the retina seem to represent precursors or components of hard exudates. Turgut *et al*.^22^ investigated the causes of HRF in SD OCT excluding DME and RVO. Vujosevic *et al*.^23^ evaluated the presence of HRF in diabetic patients with NDR or NPDR. Vujosevic *et al*.^24^ evaluated hyperreflective retinal spots (HRS) in normal and diabetic patients on linear B-scans and corresponding *en face* images of SD OCT. Recently, however, Davoudi *et al*.^15^ investigated whether lipid serum levels were associated with the presence of HRF and macular thickness in SD OCT images. Frizziero *et al*.^25^ concluded that hyperreflective spots increase in number with increasing central subfield thickness and could be considered a new clinical biomarker of intraretinal inflammation in patients affected by macular edema secondary to irradiation for uveal melanoma. Other studies mainly focused on the presence and distribution of foci, where these foci might be associated with certain retinopathy^26, 27^. Uji *et al*.^27^ studied the association between hyper-reflective foci in outer retina, status of the photoreceptor layer, and visual acuity in diabetic macular edema and concluded the presence of hyper-reflective foci in the outer retina is closely associated with the disruption of ELM and inner segments/outer segments (IS/OS) in SD OCT images and decreased vision acuity (VA) in DME. Vujosevic *et al*.^28^ assessed early changes in intraretinal HRs after anti-vascular endothelial growth factor treatment in center-involving diabetic macular edema. The statistical results showed a significant decrease in HRS in the retina after anti-vascular endothelial growth factor treatment.

We systematically analyzed the existing studies of HRF and HEs in patients with DME and concluded that increasing numbers of hyper-reflective regions seem to be associated with an increased risk of visual loss. Furthermore, most hyper-reflective foci may be taken as the precursors of HEs in patients with DR, indicating an association in disease phenotypes between hyper-reflective foci and hard exudates. Due to their density and opacity, hard exudates are observed as hyper-reflective lesions in SD OCT B-scans; it is therefore a challenging task to study the regions where hyper-reflective foci are taken as hard exudates on SD OCT images and to explore the potential discriminant indicators of the severity of diabetic retinopathy.

**Figure 1:**
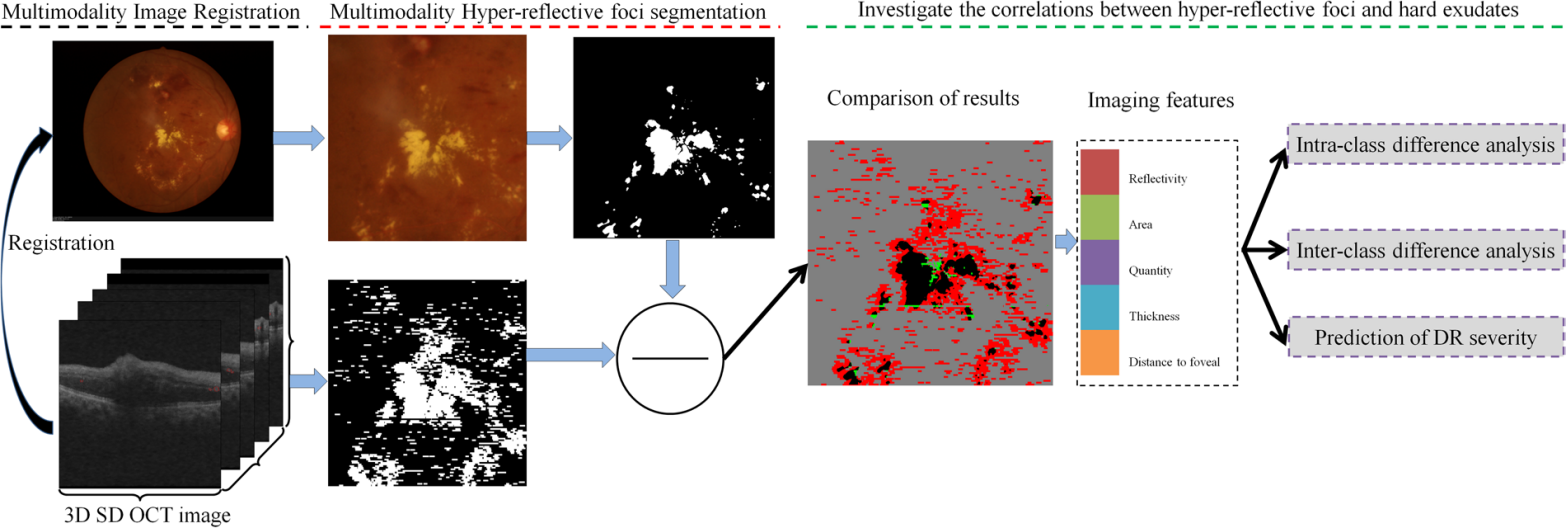
Diagram showing the processing pipeline of our multimodality analysis method.

In this paper, we investigate whether hyper-reflective foci are associated with the presence of hard exudates in patients with NPDR and PDR on SD OCT images (as shown in Fig. 1). A set of quantitative imaging features characterizing their size, shape, amount and reflectivity were automatically extracted from SD OCT images. The evaluation of the association between hyper-reflective foci and hard exudates using SD OCT imaging features in patients with different stages of DR may aid in the discussion of a possible origin for hyper-reflective foci and the evaluation of the therapy outcome. However, to the best of our knowledge, there is no published report that accurately investigates the associations between hyper-reflective foci and hard exudates using SD OCT images.

## Materials and Methods

### Patients

In this prospective study, nineteen eyes from 15 patients with PDR and fourteen eyes from 11 patients with NPDR, where all patients were diagnosed by an experienced ophthalmologist using CFP and fundus fluorescein angiography (FFA), were enrolled at the Department of Ophthalmology, the First Affiliated Hospital with Nanjing Medical University, China. All patients underwent complete ophthalmic examination: CFP, FFA and OCT. OCT examinations were performed using Zeiss SD OCT. All the SD-OCT scans were acquired using an instrument that produced an imaging volume with dimensions of 6 (horizontal) × 6 (vertical) × 2(axial) mm (512, 128, and 1024 voxels in each direction). The raw data produced by the SD-OCT instrument were imported into the vendor’s proprietary software for analysis and reconstruction (Zeiss Research Browser, version 6.0.2.81; Carl Zeiss Meditec, Inc.). All the data processing and methods were later implemented and carried out using Matlab (The MathWorks, Inc., Natick, MA, USA). The research was approved by the institutional Review Board of the first Affiliated Hospital with Nanjing Medical University (Jiangsu Province Hospital). All state and local laws were abided by, and all subjects signed the informed consent.

Inclusion criteria for this study were patients diagnosed as PDR or NPDR by a clinical ophthalmologist. All patients with CFPs and OCT images of sufficient quality were included in this analysis. In cases with very low visual acuity, it is difficult to obtain high-quality images because of poor eye fixation.

### Agreement assessment of hard exudates in CFP and SD OCT images

**Figure 2:**
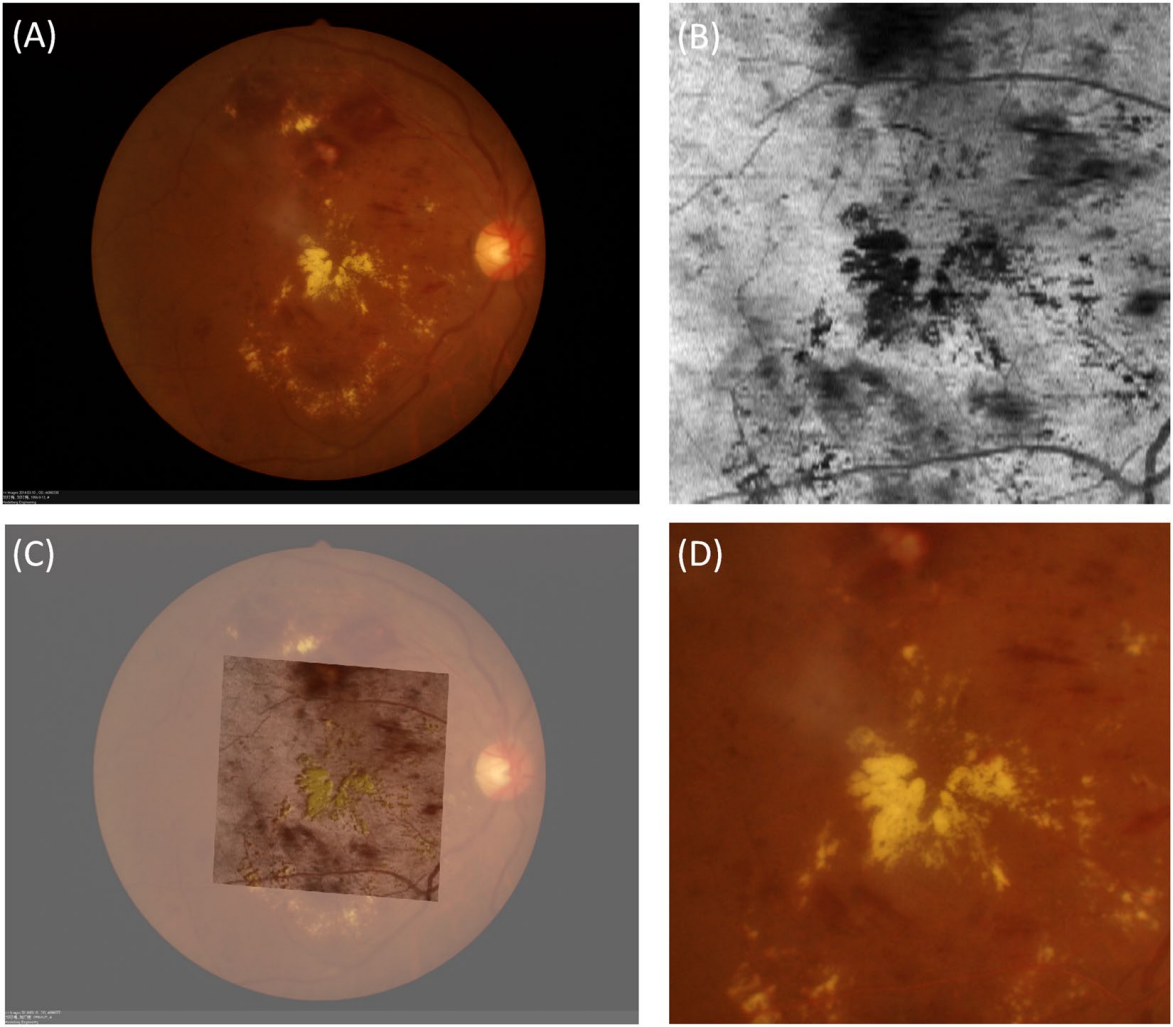
Registration of CFP and SD OCT *en face* image. (**A**) Original CFP image. (**B**) SD OCT *en face* image generated by projecting a region between the IS/OS and BM boundaries^33^, where the *en face* image can better highlight blood vessels. (**C**) SD OCT *en face* image is superposed on the CFP image. (**D**) The cropped CFP image.

Owing to the reflective-based properties of the various cellular layers in SD OCT, differentiation of hard exudates from other hyper-reflective lesions within the retinal layers remains a challenging task. To assess the agreement of hard exudates in both CFP and SD OCT images, overlaps of the two imaging types were generated by the semi-automatic registration method^29^. Retinal vessel intersection points taken as landmarks were used to calculate the transformation model; then, based on the transformation model, the CFP images were registered with the SD OCT *en face* images and cropped for further use (Fig. 2).

We designed a saliency detection method at different scales to segment hard exudate regions from the cropped CFP images. Its primary principle is that the saliency is estimated by the local contrast of an image region with respect to its neighborhood at different scales. The estimation is generated by calculating the distance between the feature vectors of the pixels of an image sub-region and the feature vector of the pixels of its neighborhood. At a given scale, the saliency for a pixel position (*i*, *j*) is defined as:

1 $${C}_{i,j}=\Vert {v}_{1}-{v}_{2}\Vert $$

where *v*_1_ is the feature vector of the local image region, and *v*_2_ is the feature vector of its neighborhood region. In our work, we used CIELab color space to generate *v*_1_ = [*L*_1_, *a*_1_, *b*_1_]^T^ and *v*_2_ = [*L*_2_, *a*_2_, *b*_2_]^T^. The final saliency is estimated as a sum of saliency across the scales *L*:

2 $${S}_{i,j}=\sum _{L}{C}_{i,j}$$

Finally, the hard exudate regions are detected by segmenting the final saliency map with the threshold method. The pseudo hard exudates are manually removed by an experienced clinical ophthalmologist from the Department of Ophthalmology, guaranteeing that the segmentation results can meet the subsequent statistical analyses.

In retinal SD OCT images of patients with PDR or NPDR, HEs are observed as hyper-reflective foci. To segment the HRFs from the SD OCT image, we developed a novel algorithm, which first segments both the nerve fiber layer (NFL) and IS/OS boundaries and then constructs a narrow-band including HRFs. The details of this algorithm are as follows: A bilateral filter is applied to reduce image noise before follow-up processing^30^. Considering that NFL and OS have similar intensity with HRF, we intend to segment the down boundary of the NFL and OS layer to limit the regions where foci locate. A two-dimensional (2D) layer segmentation method is adopted to segment these two boundaries^31^. Then, we use the self-adaption threshold and region growing to extract HRF. However, our method is not robust enough because there might be regions belonging to the background that almost have the same intensity as the target foci while being located in the limited region. Therefore, the poor segmentation results are supposed to be corrected by hand. We try our best to guarantee the precision of foci segmentation to ensure the subsequent statistical analyses.

Using the regions of HE segmented from the cropped CFP images, the corresponding HE regions in the SD OCT *en face* images were generated. Subsequently, we evaluated the agreement of hard exudates in both CFP images and SD OCT *en face* images.

### Characteristic analysis of hyper-reflective foci and hard exudates in SD OCT images

To correlate the HRF and HEs in SD OCT images, we investigated the inclusion of 5 comprehensive quantitative imaging features for lesions in SD OCT B-scan images. A set of 3 features characterizing the extent and shape of lesions were extracted from lesion regions in SD OCT B-scan images, including area, amount and reflectivity. 2 features were included to describe their distribution patterns in different stages of the disease, including average distance to macular fovea of all lesions, and average height of all axial directions in the lesion regions. The average distance to macular fovea is defined as the Euclidean distance between a lesion centroid and the foveal center manually determined by the lowest point in the foveal depression on both horizontal and vertical planes. Based on these quantitative features, we analyzed the relationship between HRF and HEs using correlation coefficient (cc), *p*-value, and statistical distribution.

### Evaluation of the severity of DR based on the imaging features

We utilized the libsvm tool package^32^, which can deal with multi-class problems to construct our evaluation model and predict the severity of diabetic retinopathy as NPDR or PDR. Here, eyes with NPDR and PDR were taken as positive and negative samples, respectively. Differences in hyper-reflective foci and hard exudates between NPDR and PDR were analyzed by comparing a set of features in previous sections. We used the 8 combinations of the normalized imaging features to build the predicted model and investigate the importance of the observed imaging features for predicting the severity of diabetic retinopathy. Because the number of samples in our experiment was not large enough, leave-one-out cross validation was adopted to evaluate the evaluation ability of our model. The regularization parameter *c* and the Radial basis kernel width parameter *g* were optimized by a grid search strategy in the libsvm tool package. We used Accuracy (*Acc*), Sensitivity (*Sen*), Specificity (*Spe*) and Matthew correlation coefficient (*MCC*) to assess the predicted ability of our model, defined as follows:

3 $$\{\begin{array}{rcl}Sen & = & \frac{TP}{TP+FN}\\ Spe & = & \frac{TN}{TN+FP}\\ Acc & = & \frac{TP+TN}{TP+TN+FP+FN}\\ MCC & = & \frac{(TP\times TN)-(FP\times FN)}{\sqrt{(TP+FP)(TP+FN)(TN+FP)(TN+FN)}}\end{array}$$

where *TP*, *FP*, *TN* and *FN* are the true positive, false positive, true negative, and false negative, respectively. Evaluation indexes described by formula (3) can also be regarded as confidences when predicting the severity of a patient with DR.

## Results

**Figure 3:**
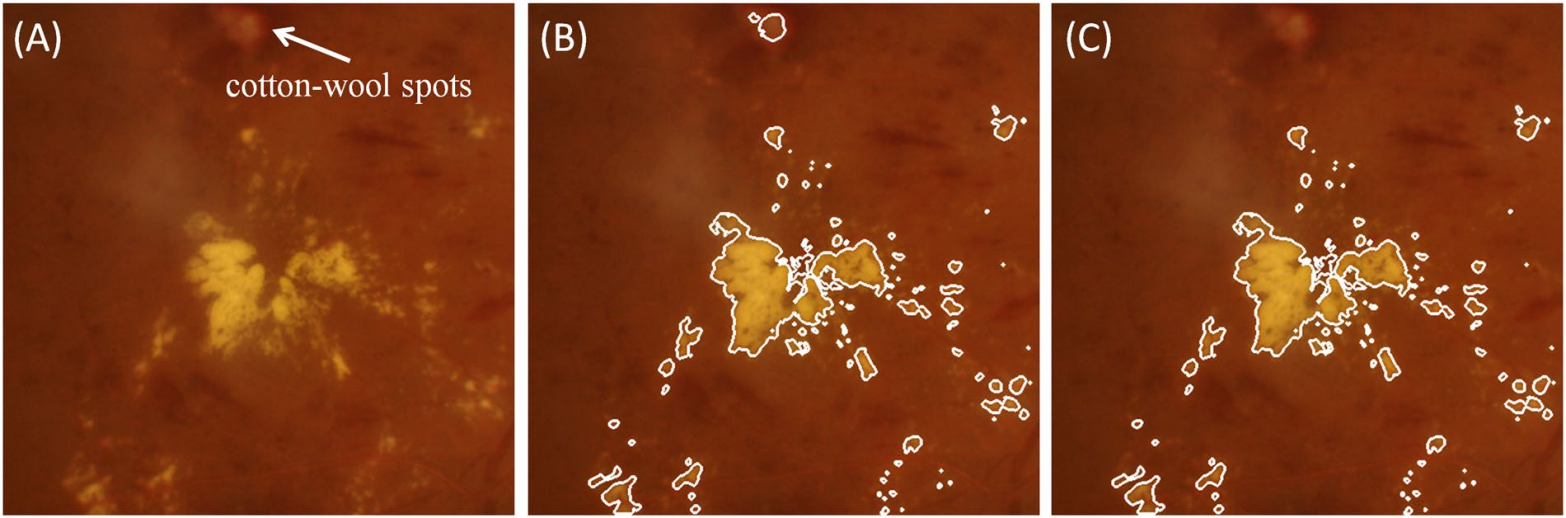
Example of hard exudates segmentation. (**A**) The cropped CFP image. (**B**) Automatic segmentation is superposed on the cropped CFP image, where the white line represents the outline of the hard exudates. (**C**) The corrected segmentation of hard exudates.

**Figure 4:**
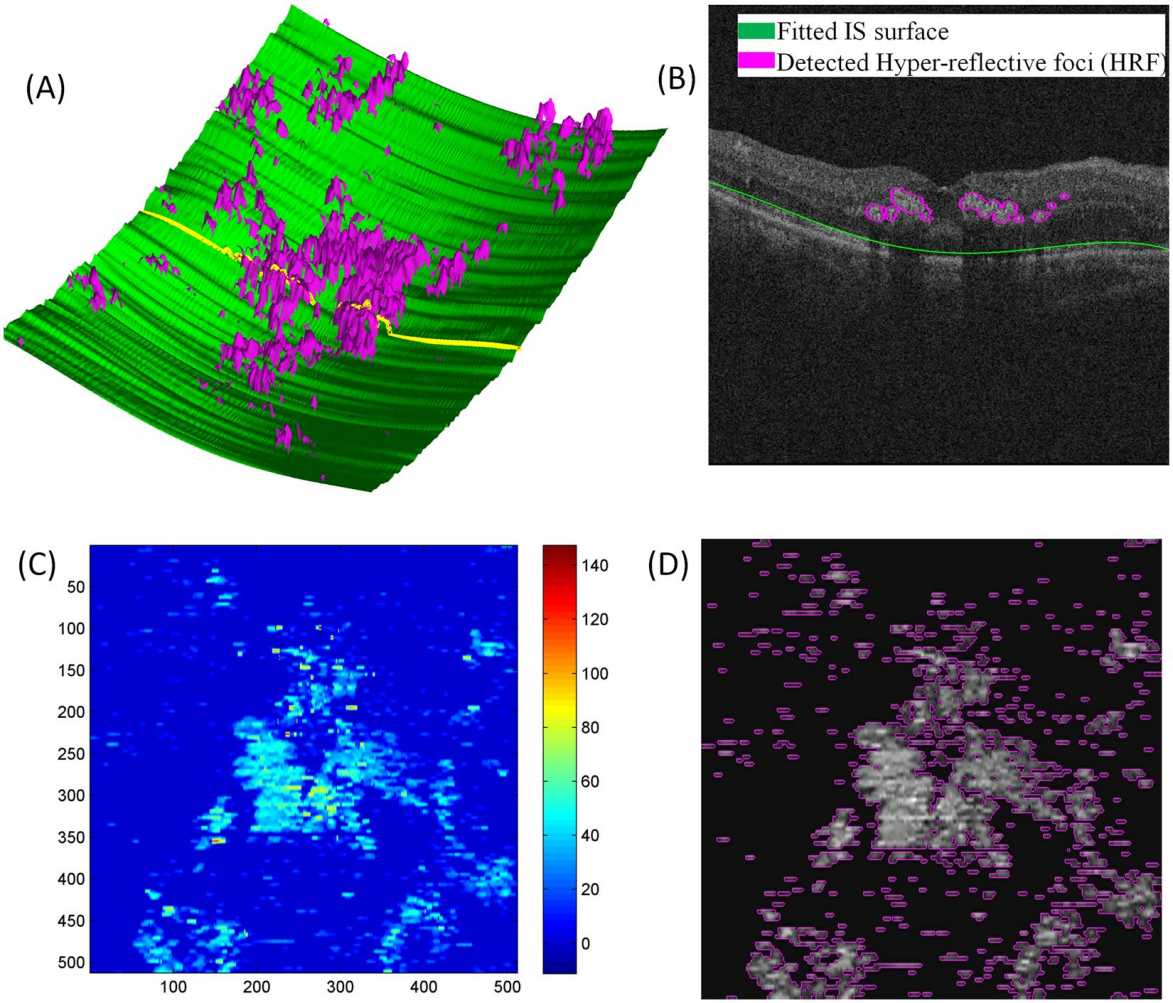
Hyper-reflective foci segmentation, (**A**) Hyper-reflective foci 3D surface view, where green and pink represent the fitted IS surface and the detected hyper-reflective foci, respectively. (**B**) Results from hyper-reflective foci segmentation in an example of a B-scan, corresponding to the yellow line in (**A**). (**C**) Generated hyper-reflective foci topographic height map and (**D**) Topographic height map segmentation.

Fourteen eyes from 14 patients with NPDR and nineteen eyes from 19 patients with PDR were used to perform linear regression analysis in this study. Figure 3 showed an example of the hard exudates segmentation on CFP image, where several pseudo hard exudates (such as cotton-wool spots) appear on the cropped CFP image. The coarse segmentation results were manually corrected by an experienced clinical ophthalmologist (Fig. 3(C)). For the hyper-reflective foci on SD OCT images, an example of three-dimensional (3D) segmentation, the corresponding B-scan, topographic height map and height segmentation is displayed as Fig. 4.

### Results of agreement evaluation for hard exudates

**Figure 5:**
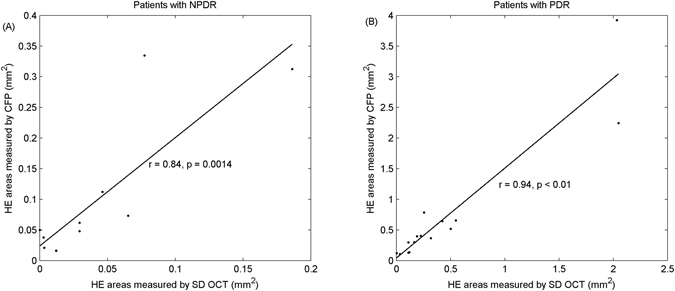
Results of agreement evaluation for hard exudates between CFP and SD OCT. Correlations between HE areas measured by SD OCT and CFP at NPDR and PDR are displayed as (**A**) and (**B**), respectively.

Correlations between HE areas measured by SD OCT and CFP in both NPDR and PDR were 0.84 and 0.94, respectively, while their corresponding *p*-values in correlations were significant (with *p* = 0.0014 and *p* < 0.01, respectively), as shown in Fig. 5. The slopes of the fitting curves were 1.77 and 1.47 for patients with NPDR and PDR, respectively. The results demonstrated that the proposed segmentation method for HE detection in SD OCT has good performance when compared to CFP and that SD OCT may provide a novel method to analyze HEs.

**Figure 6:**
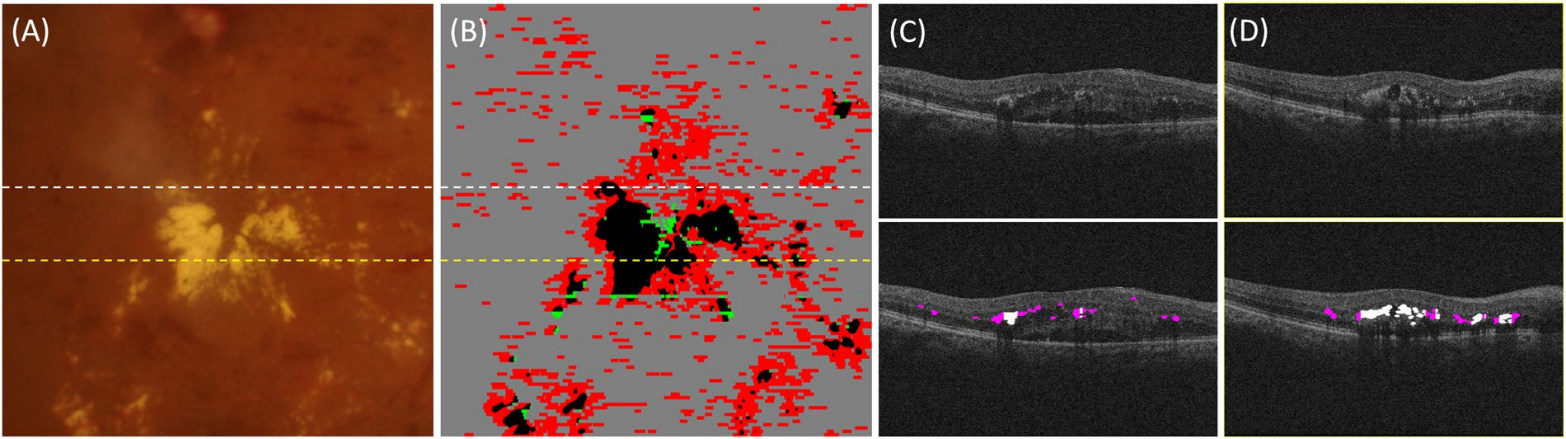
The combined analysis of SD OCT B-scan and aligned CFP. (**A**) The cropped CFP images, where hyper-reflective regions represent hard exudates. (**B**) The comparison of HE segmentation results on CFP and hyper-reflective foci results on SD OCT. The black regions are hard exudates measured by CFP and SD OCT. The green regions measured by CFP are hard exudates that are missed by SD OCT. The red regions represent hyper-reflective foci on SD OCT, where these foci could not be identified in the corresponding CFP. (**C**) and (**D**) SD OCT B-scan images correspond to the white and yellow dotted line, respectively, where the pink regions and the white regions are hyper-reflective foci and hard exudates, respectively.

In the combined analysis of the SD OCT B-scan and registered CFP (Fig. 6), hyper-reflective foci were mainly found in the ONL and OPL, while small foci may appear in the inner nuclear layer (INL) and inner plexiform layer (IPL). On the SD OCT B-scan, the foci with higher altitude in the axial direction accumulated at the border of ONL and OPL, which were taken as HEs. Furthermore, we also found that larger foci showed a shadowing phenomenon in the inner retinal layer. According to statistical analysis of the HE regions detected by the aligned CFP, their reflectivity is close to the reflectivity of the foci. This combined analysis suggests that distinguishing HEs from hyper-reflective foci in SD OCT images could be a challenging task.

### Results of characteristic analysis of hyper-reflective foci and hard exudate

**Figure 7:**
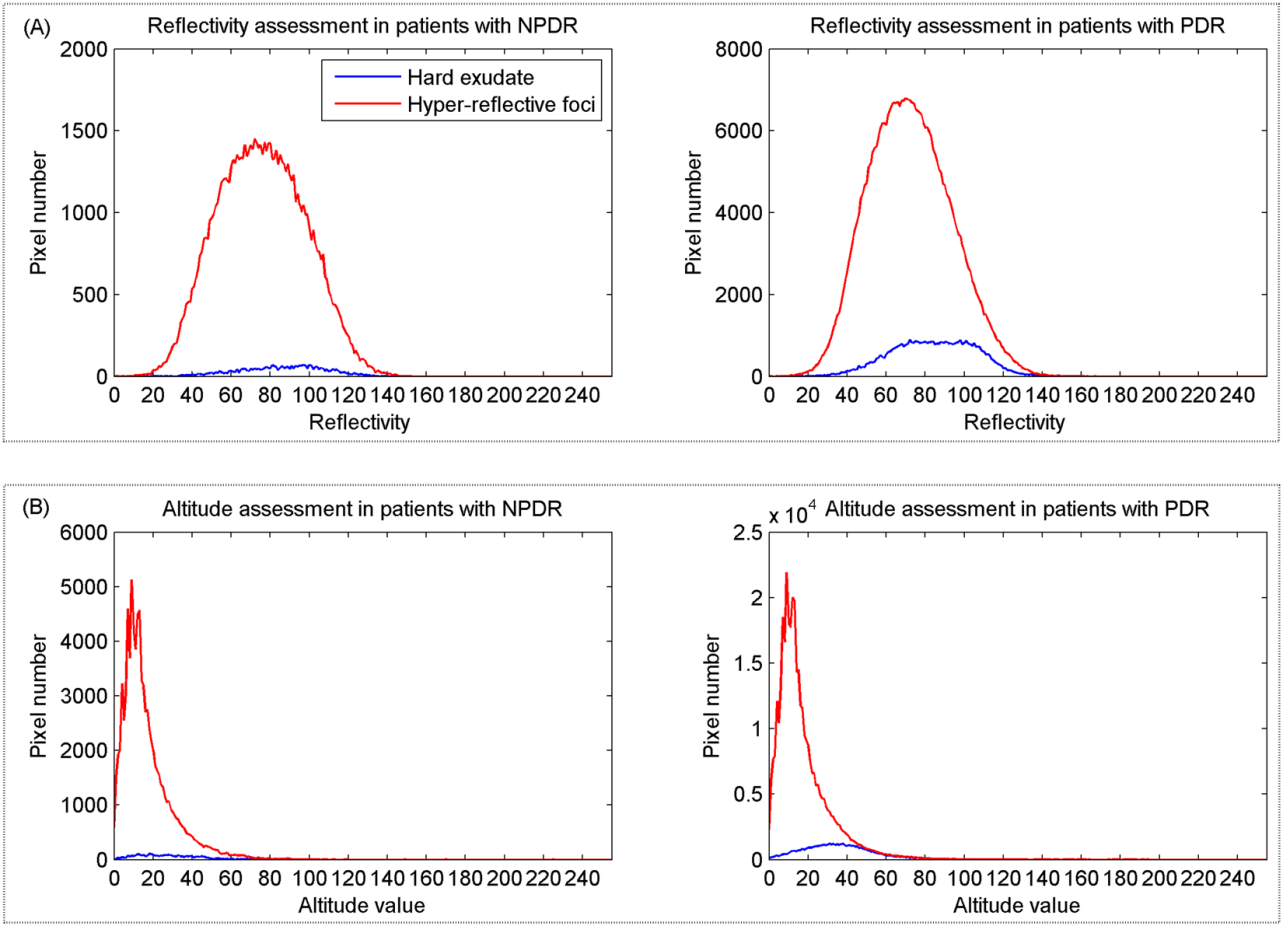
Results of the reflectivity and altitude distribution in SD OCT. (**A**) Reflectivity assessment of hyper-reflective foci and hard exudate in patients with NPDR and PDR. (**B**) Results of the altitude distribution of hyper-reflective foci and hard exudate in patients with NPDR and PDR.

In this section, a set of 5 features were characterized to assess associations between hyper-reflective foci and hard exudates, including altitude in the axial direction, reflectivity distribution, amount, area, and average distance to macular fovea per lesion. Figure 7(A) showed that the reflectivity of hyper-reflective foci (excluding hard exudates) and hard exudates in patients with NPDR and PDR are concentrated around 80, indicating that they have similar reflectivity distribution. However, the distribution of their altitude could be an interesting factor for differentiating hard exudates from hyper-reflective foci. The altitude of hard exudate (with average altitude of 53.5 μm and 69.6 μm in both NPDR and PDR, respectively) is higher than hyper-reflective foci (excluding hard exudates; with average altitudes of 32.9 μm and 33.6 μm in both NPDR and PDR, respectively), in particular in PDR, as shown in Fig. 7(B). For the area and amount, the correlations between hyper-reflective foci (including hard exudates) and hard exudates in patients with NPDR and PDR are relatively high (0.86, 0.68, 0.65 and 0.62, respectively), with statistical significance (all p < 0.05). The average distance to macular fovea for hard exudates and hyper-reflective foci (including hard exudates) in patients with NPDR and PDR have relatively low correlations (Fig. 7(C)).

On the other hand, we evaluated the difference in hyper-reflective foci and hard exudates between NPDR and PDR, considered as inter-class difference. For hard exudates, the differences in average altitude (0.45 ± 0.33 vs. 0.64 ± 0.25), average reflectivity (0.60 ± 0.28 vs. 0.65 ± 0.21) and average distance to foveal center (0.38 ± 0.29 vs. 0.45 ± 0.21) between the NPDR and PDR stages are relatively insignificant (all p > 0.05), while the differences in average area (0.02 ± 0.03 vs. 0.23 ± 0.32) and average amount (0.14 ± 0.14 vs. 0.48 ± 0.26) are relatively significant (all p < 0.05), as shown in Fig. 9(A). Figure 9(B) displayed the analysis of the difference in hyper-reflective foci (excluding hard exudates) between NPDR and PDR, where the differences in average area (0.16 ± 0.15 vs. 0.44 ± 0.30) and average amount (0.21 ± 0.19 vs. 0.56 ± 0.26) are relatively significant (all p < 0.05) and the difference in average altitude (0.35 ± 0.25 vs. 0.44 ± 0.23), average reflectivity (0.64 ± 0.26 vs. 0.54 ± 0.31) and average distance to foveal center (0.62 ± 0.27 vs. 0.67 ± 0.13) are relatively insignificant (all p > 0.05). The difference results demonstrated that the average area and average amount may be taken as promising biomarkers to predict the severity of diabetic retinopathy.

### Results of evaluation of DR severity

Table 1 displayed the results of evaluation indexes in each leave-one-out process where parameters of the model were optimally ensured. We observed that the evaluation indexes can reach the highest values (Acc of 75.76%, 78.79% and 75.76% in three different lesions) with the combination of average area and average amount as inputs, and with other features appended, the effect of the evaluation model went down overall, which suggested that these two features could be taken as potential biomarkers to predict the severity of DR, as analyzed in the previous section. Moreover, the accuracy of the predicted model based on HRF (excluding HEs) is better than that of the predicted model based on HEs. It is possible that several eyes with NPDR and PDR do not have HEs and are therefore difficult to distinguish.

**Table 1:** Results of evaluation indexes under different feature combinations based on HEs, HRF (excluding HEs) and HRF, Unit: %.

	HEs				HRF (excluding HEs)				HRF (including HEs)			
	*Sen*	*Spe*	*Acc*	*MCC*	*Sen*	*Spe*	*Acc*	*MCC*	*Sen*	*Spe*	*Acc*	*MCC*
1, 2	**92.86**	**63.16**	**75.76**	**56.66**	**92.86**	**68.42**	**78.79**	**61.28**	**85.71**	**68.42**	**75.76**	**53.73**
1, 2, 3	92.86	63.16	75.76	56.66	92.86	68.42	78.79	61.28	85.71	68.42	75.76	53.73
1, 2, 4	78.57	68.42	72.73	46.47	92.86	68.42	78.79	61.28	85.71	68.42	75.76	53.73
1, 2, 5	57.14	68.42	63.64	25.56	78.57	63.16	69.70	41.42	78.57	63.16	69.70	41.42
1, 2, 3, 4	57.14	68.42	63.64	25.56	85.71	68.42	75.76	53.73	78.57	68.42	72.73	46.47
1, 2, 3, 5	57.14	63.16	60.61	20.15	71.43	57.89	63.64	29.11	71.43	63.16	66.67	34.20
1, 2, 4, 5	50.00	68.42	60.61	18.63	71.43	63.16	66.67	34.20	71.43	63.16	66.67	34.20
1, 2, 3, 4, 5	50.00	68.42	60.61	18.63	71.43	57.89	63.64	29.11	64.29	63.16	63.64	27.14

*No. 1–5 represent average area, average amount, average distance, average altitude and average reflectivity, respectively.

## Discussion

The aim of this study is to investigate the associations between hyper-reflective foci and hard exudates in patients with diabetic retinopathy (including NPDR and PDR) based on extracting a set of quantitative features that characterize the phenotypes associated with them in SD OCT images. Although previous work^18–28^ studied changes in hard exudates or hyper-reflective foci in patients with diabetic macular edema (DME) using SD OCT images, they did not assess the relationship between hard exudates and hyper-reflective foci in SD OCT images or evaluate DR severity using a set of quantitative imaging features. Additionally, our work appears to be a novel approach to discuss the correlations between hyper-reflective foci and hard exudates; and a model is built to objectively predict the severity of DR based on quantitative imaging features. The results showed that the correlations between hyper-reflective foci and hard exudates in patients with NPDR and PDR by SD OCT were good, which would allow for better understanding of the pathogenesis and investigating the causes of hyper-reflective foci in SD OCT images.

From the experimental results, we presented several meaningful conjectures: (1) In the combined analysis of SD OCT B-scan and aligned CFP, we found that small hyper-reflective foci in SD OCT were not observed by CFP. There are three possible explanations: First, the size of hyper-reflective foci might be too small to accumulate into visible lesions. Second, the axial thickness of hyper-reflective foci is smaller than the axial thickness of hard exudates (Figs 4(C) and 7(B)), indicating that the axial thickness of hyper-reflective foci is not thick enough to be identified in CFP. Third, the imaging resolution of SD OCT (1~10 μm) is higher than the color photograph fundus imaging. (2) Hyper-reflective foci might accumulate into large, thick and visible lesions with time and therefore might be considered hard exudates. In addition, the reflectivity distribution of hard exudates and hyper-reflective foci are similar as shown in Fig. 7(A), indicating they have the same pathogenesis or they have the same origin. (3) Hyper-reflective foci may aid in early detection of diabetic retinopathy or early diagnosis of diabetic retinopathy for non-professional clinicians.

A statistical analysis of image reflectivity (shown in Fig. 7(A)) showed that hyper-reflective foci and hard exudates have a similar reflectivity distribution in both the NPDR and PDR stages. The findings suggested that the differentiation of HEs from hyper-reflective foci within retinal layers remains difficult due to the reflectivity-based imaging principle of SD OCT. Results of agreement evaluation for hard exudates showed good correlation (*r* = 0.84 and *r* = 0.94 in both NPDR and PDR, respectively) between HEs in CFP and corresponding hyper-reflective foci in SD OCT, indicating that hard exudates may be represented by hyper-reflective foci in SD OCT images. To investigate the association between hyper-reflective foci and hard exudates in SD OCT images, we try to automatically extract a set of imaging biomarkers from SD OCT images. Their characteristic analyses displayed that the axial altitude may be a factor that differentiates hard exudates from hyper-reflective foci in SD OCT images, as shown in Fig. 7(B), and the average area and the average amount may be potential discriminative indicators of the severity of diabetic retinopathy (Fig. 9).

**Figure 8:**
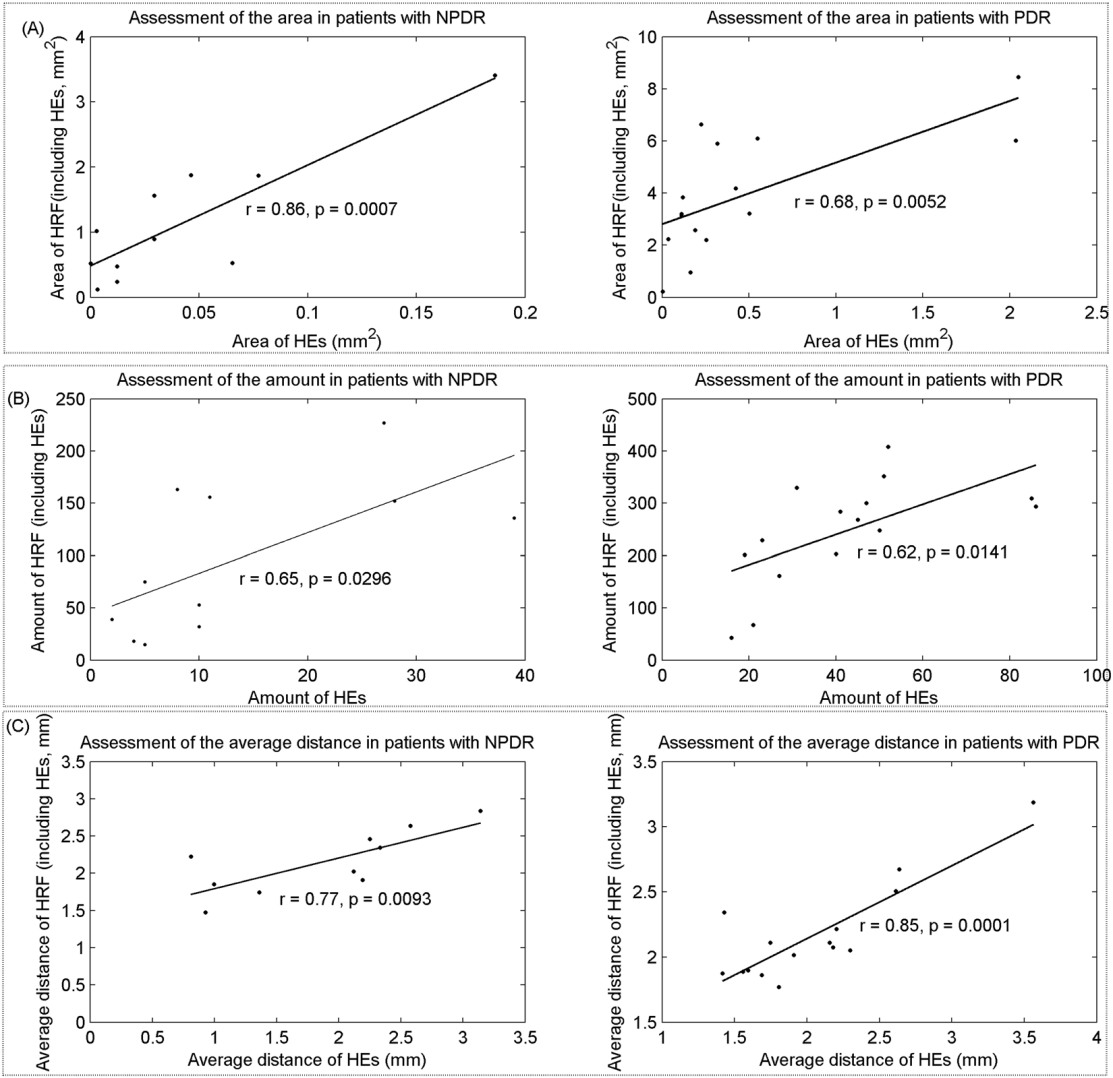
(**A**) Area assessment of hard exudate and hyper-reflective foci (including hard exudates) in patients with NPDR and PDR on SD OCT, (**B**) Results from the amount assessment of hard exudate and hyper-reflective foci (including hard exudates) in patients with NPDR and PDR on SD OCT. (**C**) Average distance to macular fovea for hard exudate and hyper-reflective (including hard exudates) in patients with NPDR and PDR.

In early stage of diabetic retinopathy, hyper-reflective foci may represent small intraretinal proteins or lipoprotein extravasation after breakdown of the blood-retinal barrier in the initial stages of hard exudate development^6, 8, 19^. With the thickening of hyper-reflective foci to a certain extent, these foci, initially isolated and small, may accumulate into clinically visible hard exudates^8^. This finding was supported by our results that showed the positive correlation in area and amount between hard exudates and hyper-reflective foci at different stages of diabetic retinopathy, with statistical significance (all p < 0.05), as shown in Fig. 8.

**Figure 9:**
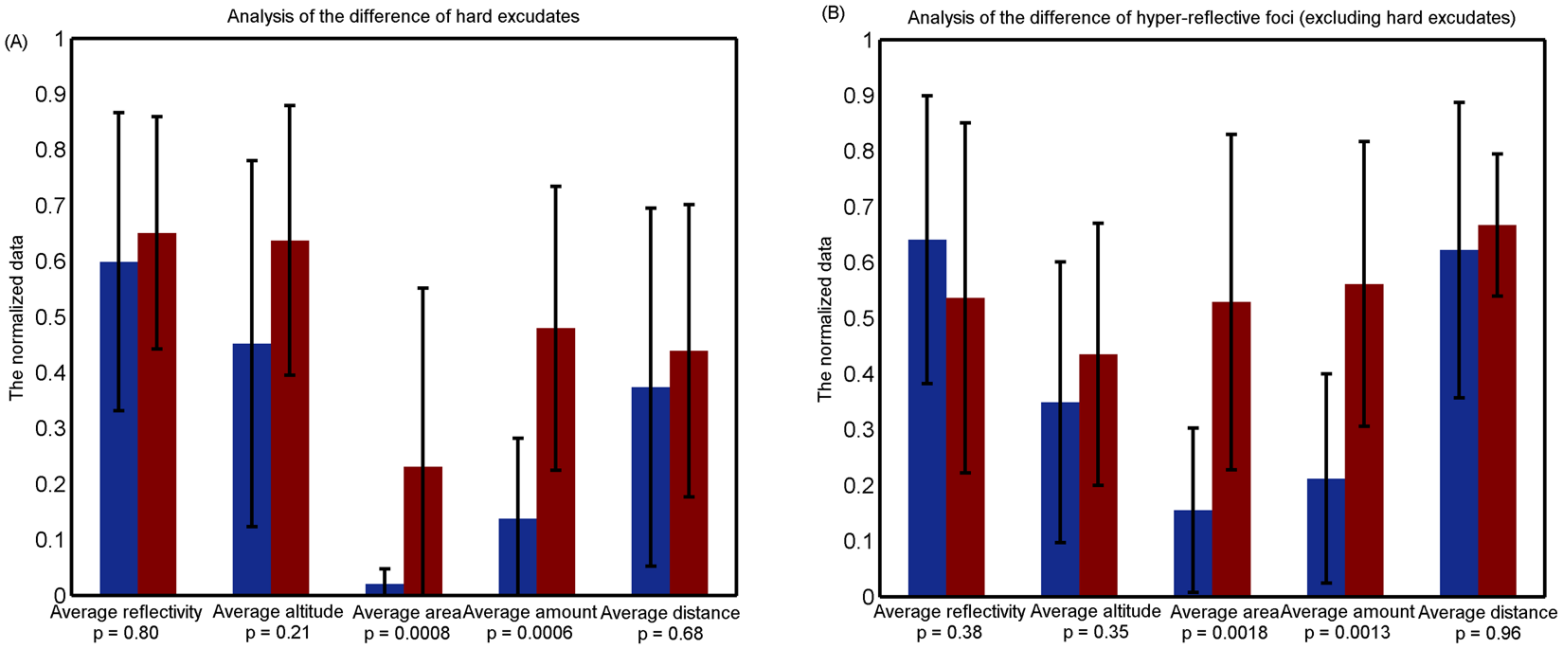
Results of analysis of the difference. (**A**) Results of the difference in hard exudates between NPDR and PDR in SD OCT. (**B**) Results of the difference in hyper-reflective foci (excluding hard exudates) between NPDR and PDR in SD OCT.

For hyper-reflective foci in SD OCT images, the average area and the average amount increase with the progression of diabetic retinopathy; however average altitude, average distance and average reflectivity increase slowly, as shown in Fig. 9(B). By tracking the changes of these imaging biomarkers, we built a model to objectively predict the severity of diabetic retinopathy (NPDR or PDR). Furthermore, the results of statistical analyses of hyper-reflective foci indicated that their quantitative features in different stages of diabetic retinopathy are statistically significant. Consequently, clinical ophthalmologists may diagnose and follow up on diabetic retinopathy based on SD OCT imaging features of hyper-reflective foci.

Regarding the relationship of hyper-reflective foci and hard exudates, hyper-reflective foci, with their larger size and back shadowing, formed confluent deposits in the inner part of the outer retinal layers and appeared clinically as hard exudates based on color fundus photography. Instead, hyper-reflective foci, which are small and exhibit no back shadowing, may be considered as aggregates of activated microglial cells^11, 23^, the precursors of HEs, or some small retinal capillaries^24^. Therefore, using a variety of imaging modalities and a larger amount of study data may be useful to precisely investigate the potential origin of hyper-reflective foci.

However, our work still presented a number of limitations. Errors in the segmentation of hard exudates and hyper-reflective foci may be caused by some lesions, such as cotton-wool spots, speckle noises or blood vessels, resulting in possible inaccuracies in the correlation analysis. However, all segmentation and registration for the images included in this study were visually reviewed and considered adequate for this analysis by an experienced ophthalmologist. Another limitation of this study is that the sample data are not comprehensive enough to perform a systematic investigation, although several interesting findings are statistically significant. In future research, we will try to collect more data to continue our study of the association between hyper-reflective foci and visual loss and progression prediction.

## References

[1] Congdon NG, Friedman DS, Lietman T (2003). Important causes of visual impairment in the world today. JAMA.

[2] Fong DS, Aiello LP, Ferris FL, Klein R (2004). Diabetic retinopathy. Diabetes Care.

[3] Chen L, Magliano DJ, Zimmet PZ (2012). The worldwide epidemiology of type 2 diabetes mellitus –present and future perspectives. Nat Rev Endocrinol.

[4] Cheung N, Wong TY (2008). Diabetic retinopathy and systemic vascular complications. Prog Retin Eye Res.

[5] Chew EY (1996). Association of elevated serum lipid levels with retinal hard exudate in diabetic retinopathy: Early Treatment Diabetic Retinopathy Study Report 22. Archives of Ophthalmology.

[6] De BU, Sacconi R, Pierro L, Lattanzio R, Bandello F (2014). Optical coherence tomographic hyperreflective foci in early stages of diabetic retinopathy. Retina.

[7] Cusick M (2003). Histopathology and regression of retinal hard exudates in diabetic retinopathy after reduction of elevated serum lipid levels. Ophthalmology.

[8] Bolz M (2009). Opthical coherence tomographic hyperreflective foci: a morphologic sign of lipid extravasation in diabetic macular edema. Ophthalmology.

[9] Yehoshua Z, Rosenfeld PJ, Gregori G, Feuer WJ (2011). Progression of Geographic Atrophy in Age-Related Macular Degeneration Imaged with Spectral Domain Optical Coherence Tomography. Ophthalmology.

[10] Zysk AM, Nguyen FT, Oldenburg AL (2007). Optical coherence tomography: a review of clinical development from bench to bedside. Journal of Biomedical Optics.

[11] Coscas G (2013). Hyperreflective dots: a new spectral-domain optical coherence tomography entity for follow-up and prognosis in exudative age-related macular degeneration. Ophthalmologica.

[12] De Niro JE, McDonald HR, Johnson RN (2014). Sensitivity of fluid detection in patients with neovascular AMD using spectral domain optical coherence tomography high-definition line scans. Retina.

[13] Shuang L, Wang B, Yin B (2014). Retinal nerve fiber layer reflectance for early glaucoma diagnosis. Journal of Glaucoma.

[14] Akashi A, Kanamori A, Nakamura M (2013). The ability of macular parameters and circumpapillary retinal nerve fiber layer by three SD-OCT instruments to diagnose highly myopic glaucoma. Investigative Ophthalmology & Visual Science.

[15] Davoudi S (2016). Optical Coherence Tomography Characteristics of Macular Edema and Hard Exudates and their association with Lipid Serum Levels in Type 2 Diabetes. Retina.

[16] de Sisternes L, Simon N, Tibshirani R, Leng T, Rubin DL (2014). Quantitative SD-OCT Imaging Biomarkers as Indicators of Age-Related Macular Degeneration Progression. Investigative Ophthalmology & Visual Science.

[17] Niu SJ, de Sisternes L, Chen Q, Rubin DL, Leng T (2016). Fully Automated Prediction of Geographic Atrophy Growth Using Quantitative Spectral-Domain Optical Coherence Tomography Biomarkers. Ophthalmology.

[18] De Benedetto U, Sacconi R, Pierro L, Lattanzio R, Bandello F (2015). Optical coherence tomographic hyperreflective foci in early stages of diabetic retinopathy. Retina.

[19] Lammer J (2014). Detection and analysis of hard exudates by polarization-sensitive Optical Coherence Tomography in Patients with Diabetic Maculopathy. Investigative Ophthalmology & Visual Science.

[20] Ota M (2010). Optical coherence tomographic evaluation of foveal hard exudates in patients, with diabetic maculopathy accompanying macular detachment. Ophthalmology.

[21] Deák GG (2010). Effect of retinal photocoagulation on intraretinal lipid exudates in diabetic macular edema documented by optical coherence tomography. Ophthalmology.

[22] Turgut B, Hakan Y (2015). The Causes of Hyperreflective Dots in Optical Coherence Tomography Excluding Diabetic Macular Edema and Retinal Venous Occlusion. Open Ophthalmology Journal.

[23] Vujosevic S (2013). Hyperreflective intraretinal spots in diabetics without and with nonproliferative diabetic retinopathy: an *in vivo* study using spectral domain OCT. Journal of diabetes research.

[24] Vujosevic, S. *et al*. Hyperreflective retinal spots in normal and diabetic eyes: B-Scan and En Face Spectral Domain Optical Coherence Tomography Evaluation. Retina, doi:10.1097/IAE.0000000000001304 (2016). 10.1097/IAE.0000000000001304 27668929

[25] Frizziero L (2016). Hyperreflective intraretinal spots in radiation macular edema on spectral domain optical coherence tomography. Retina.

[26] Ogino K (2012). Characteristics of optical coherence tomographic hyperreflective foci in retinal vein occlusion. Retina.

[27] Uji A (2012). Association between hyperreflective foci in the outer retina, status of photoreceptor layer, and visual acuity in diabetic macular edema. American Journal of Ophthalmology.

[28] Vujosevic S (2016). Hyperreflective retinal spots and visual function after anti-vascular endothelial growth factor treatment in center-involving diabetic macular edema. Retina.

[29] Niu, S. J., Chen, Q., de Sisternes, L., Rubin, D. L. Registration of SD-OCT en-face images with color fundus photographs based on local patch matching. Proceedings of the Ophthalmic Medical Image Analysis First International Workshop, OMIA 2014, Held in Conjunction with MICCAI 2014, Iowa Research Online, 25–32 (2014).

[30] Chen Q, de Sisternes L, Leng T, Rubin DL (2015). Application of improved homogeneity similarity-based denoising in optical coherence tomography retinal images. Journal of Digital Imaging.

[31] Chiu SJ (2010). Automatic segmentation of seven retinal layers in SDOCT images congruent with expert manual segmentation. Optics Express.

[32] Chang CC, Lin CJ (2011). LIBSVM: A library for support vector machines. Acm Transactions on Intelligent Systems & Technology.

[33] Chen Q (2013). Semi-automatic geographic atrophy segmentation for SD-OCT images. Biomedical Optical Express.

